# 

**Published:** 1899-02-11

**Authors:** 


**Notices of Births, Deaths, and Marriages** are inserted in this column at a charge of 2s. 6d. for an announcement not exceeding four lines.

### NOTICE TO CORRESPONDENTS.

All MS., Letters, Books for Review, and other matters intended for the Editor should be addressed The Editor, "The Hospital," The Hospital Building, 28 & 29, Southampton Street, Strand, W.C.
The Editor cannot undertake to return rejected MS., even when accompanied by stamped directed envelope.

**Notice to Advertisers and Subscribers.**

All Advertisements, Orders for Copies of The Hospital, and Business Communications should be addressed to **THE MANAGER (Not to the Editor),** The Hospital, The Hospital Building, 28 & 29, Southampton Street, Strand, London, W.C.
The Cost of Subscription, post free, is as follows:—Per week, 2½d.; per quarter, 2s. 8d.; per half-year, 5s. 3d.; per annum, 10s. 6d. Foreign:—Per annum, 15s. 2d., payable, in all cases, in advance.

**NOTICE.—Vol. XXIV. of "The Hospital" (April, 1898, to September, 1898), in handsome cloth binding, is now on sale at the Office of this Journal, price 7s. 6d. Cases for binding the Numbers contained in this Volume 1s. 6d. Index to Vol. XXIV., 2½d., post free.**

**The Monthly Part for February is now ready. Price 6d., by post 8d.**

### BOOKS RECEIVED.

The Scientific Press.

"The Nursing Profession: How and Where to Train." Edited by Sir Henry Burdett, K.C.B.

Cassell and Co.

"The Year Book of Treatment, 1899."

Knight and Co.

"Fry's Law of Vaccination." By A. F. Vuliamy.

T. Fisher Unwin.

"Harry Ingleby, Surgeon." By Frederick J. Webb.

Ward, Lock, and Co.

"Cage Birds." By W. T. Greene, M.A., F.Z.S.

John Wright and Co.

"The Thermal Waters of Bath." By Gilbert Bannatyne, M.D., M.R.C.P.

Crown 8vo., Paper Covers, 6d.

THE OPEN-AIR TREATMENT OF PHTHISIS.

A Visit to Falkenstein.

By RICHARD GREENE, F.R.C.P.Ed.

London: The Scientific Press (Ltd.), 28 & 29, Southampton Street, Strand, W.C.

JUST OUT. Demy 8vo., with Portrait, cloth extra, gilt, 16s.

### GEORGE HARLEY, F.R.S.;

### Or, The Life of a London Physician.

By Mrs. ALEC TWEEDIE.

"The Life of Dr. Harley is told, not as a mere chronology, but as a series of tableaux, by which the many-sided character of the subject is brought vividly before the reader. It is certainly a book to read."—*The Hospital.*

"A singularly interesting contribution to the biographical literature of the century."—*Daily Telegraph.*

London: THE SCIENTIFIC PRESS, Ltd., 28 & 29, Southampton Street, Strand, W.C.

### "THE BURDETT SERIES" OF POPULAR TEXT BOOKS ON NURSING.

Small crown 8vo., cloth. 1s each.

No. 1.—**PRACTICAL HINTS ON DISTRICT NURSING.** By AMY HUGHES.

"The whole book bristles with good advice and common sense. It should be in the hands of every district nurse."—*British Medical Journal.*

No. 2.—**THE MATRON'S COURSE.** An Introduction to Hospital and Private Nursing. By Miss S. E. ORME.

"Short practical lectures . . . readable and instructive . . . will prove useful to all who study them."—*Scotsman.*

No. 3.—**THE MIDWIVES' POCKET BOOK.** By HONNOR MORTEN, Author of "How to Become a Nurse, and How to Succeed," &c., &c.

"The little book will admirably serve its purpose."—*Glasgow Herald.*

No. 4.—**FEVERS AND INFECTIOUS DISEASES:** their Nursing and Practical Management. By WILLIAM HARDING, M.D. Ed., M.R.C.P., Lond.

"A surprising amount of terse and perspicuous information. We heartily commend the book to nurses and to mothers of families."—*Aberdeen Free Press.*

No. 5.—**NOTES ON PHARMACY AND DISPENSING FOR NURSES.** By C. J. S. THOMPSON.

"The descriptions of the various processes are clear, the rules are to the point, and the pages are full of useful information."—*The Hospital.*

No. 6.—**THE RATIONAL USE OF ANTISEPTICS IN MIDWIFERY PRACTICE.** By JAMES MORRISON, M.D., M.B., M.R.C.S., L.R.C.P. (Lond.). [*In preparation.*

No. 7.—**CARE OF CONSUMPTIVES.** By W. H. DAW, M.R.C.S., L.R.C.P., Lond.

No. 8.—**NURSING OF SICK CHILDREN.** By J. D. E. MORTIMER, M.B., F.R.C.S., L.S.A. [*In preparation.*

No. 9.—**MENTAL NURSING.** By WILLIAM HARDING, M.D., Ed., M.R.C.P., Lond. [*In preparation.*

London: The Scientific Press, Ltd., 28 & 29, Southampton Street, Strand, W.C.

